# 72. Massive Weight Gain in People with HIV (PWH) Starting Initial Antiretroviral Therapy (ART): Risk Factors and Predictive Ability of Early Weight Gain

**DOI:** 10.1093/ofid/ofab466.072

**Published:** 2021-12-04

**Authors:** Tanit Phupitakphol, Dean McEwen, Kellie Hawkins, Edward Gardner

**Affiliations:** 1 University of Colorado, Denver, CO; 2 Denver Public Health, Denver, Colorado; 3 Denver Health and Hospital Authority, Denver, Colorado

## Abstract

**Background:**

Using a clinic cohort of ART naïve PWH, we sought to understand factors associated with massive weight gain as well as to assess if early weight gain could help predict massive weight gain at two years.

**Methods:**

This was a retrospective cohort study of PWH from a large, urban clinic initiating first ART from January 2005 through March 2019, who had 21 – 27 months follow-up without ART changes, and were suppressed (HIV-RNA < 200 cps/ml) during that time. We defined massive weight gain as the top 20% of weight gainers at two years measured by percent (%) gain compared to baseline. Using bivariate and multivariate logistic regression (including factors in bivariate analysis with p< 0.20), we assessed the association of demographics, ART regimen, baseline CD4 count, HIV viral load, and body mass index (BMI) with weight gain at 2 years. We also assessed early weight gain (between 4 and 12 wks) and its association with massive weight gain at two years.

**Results:**

Of 266 PWH included (table1), the median age was 36 years (IQR 29 - 45), 9% were women, 14% black, and 43% Latino. Overall, median % weight gain at 2 years was 4% (-1.1 – 11.6) In bivariate analyses, baseline factors significantly associated with massive weight gain included lower CD4 count, higher viral load, and lower baseline BMI. In multivariate analysis the odds of having massive weight gain were higher with lower CD4 count, adjusted odds ratio (aOR) 0.8 (95% CI 0.6 – 0.9) per 100 cells/ul increase and higher viral load, aOR 2.6 (95% CI 1.4 – 4.6) per 1 log increase. Early weights were available for 217 individuals at a median of 56 days (IQR 44 – 63) after ART initiation. Early weight gain correlated with % weight gain at 2 years (R=0.58). Individuals with ≤ 3% early weight gain represented 66% of the population and had a 10% risk (14 of 144) of having massive weight gain at 2 years. In contrast, 43 individuals had > 5% early weight gain and their risk of massive weight gain at 2 years was 56% (24 of 43).

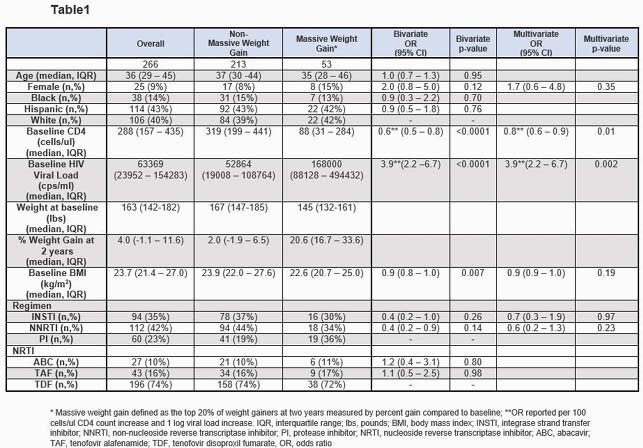

**Conclusion:**

In this real-world dataset, drug class or specific NRTI use was not associated with massive weight gain which was primarily dependent on baseline CD4 count and HIV viral load. There was a moderate correlation between early weight gain and massive weight gain at 2 years which can help with patient counseling and interventions aimed at slowing weight gain in this population.

**Disclosures:**

**All Authors**: No reported disclosures

